# Post-loss speeding and neurophysiological markers of action preparation and outcome processing in probabilistic reversal learning

**DOI:** 10.1177/17470218251333429

**Published:** 2025-04-02

**Authors:** Eugenia Kulakova, Bartosz Majchrowicz, Şiir Su Saydam, Patrick Haggard

**Affiliations:** 1 Institute of Cognitive Neuroscience, University College London, London, UK; 2 Charité – Universitätsmedizin Berlin, corporate member of Freie Universität Berlin, Humboldt Universität zu Berlin, Klinik für Psychiatrie und Psychotherapie, Campus Benjamin Franklin, Berlin, Germany; 3 DZPG (German Center for Mental Health), partner site Berlin, Germany; 4 Institute of Psychology, Jagiellonian University, Krakow, Poland; 5 Division of Psychology and Language Sciences, University College London, London, UK

**Keywords:** Probabilistic reversal learning, sequential effects, agency, outcome valence, electroencephalography, readiness potential, feedback-related negativity, P300

## Abstract

Losses and errors often slow down subsequent reaction times (RTs). This is classically explained in terms of a shift towards more cautious, therefore slow, behaviour. Recent studies of gambling, however, reported *faster* RTs following losses, so-called *post-loss speeding*, often attributing these to behavioural impulsivity arising from frustration. Here we instead investigated post-loss speeding in the context of a task that allowed behavioural adaptation and learning, namely probabilistic reversal learning (PRL). We additionally used electroencephalography (EEG) to investigate how losses influence subsequent markers of action generation (readiness potential [RP]) and outcome evaluation (feedback-related negativity [FRN] and P300). Our results confirm faster RTs after losses than after wins in PRL, thus extending post-loss speeding from gambling to cognitive contexts where learning is possible. Previous losses did not affect subsequent RP amplitudes. However, compared to wins, previous losses led to more positive FRN and more positive P300 amplitudes elicited by subsequent outcomes. Furthermore, faster RTs were associated with more negative FRN amplitudes irrespective of previous or outcome valence. We hypothesise that post-loss speeding in PRL may represent a form of signal chasing, allowing participants to behaviourally modulate neurophysiological responses and thereby potentially establish agency by influencing internal neurophysiological signals.

## Introduction

From a human perspective, the world is a place characterised by uncertainty. When acting under uncertainty, not all behavioural goals can be achieved. Therefore, errors and losses are ubiquitous to human behaviour and experience. Until recently, it was assumed that errors and losses in decision-making tasks led to *post-error slowing* (Laming, 1979; Rabbitt & Rodgers, 1977). Such effects are generally attributed to increased cognitive control required to improve subsequent performance (Botvinick et al., 2001). Recently, however, instances of post-loss *speeding* have been reported and replicated (Chen et al., 2020; Corr & Thompson, 2014; Dixon et al., 2013; Eben, Chen, et al., 2020; Eben et al., 2023; Riesel et al., 2012; Verbruggen et al., 2017; Williams et al., 2016). These typically occurred after losses in gambling tasks or other paradigms in which participants lacked agentic control over their actions’ outcomes. Post-loss speeding was thus interpreted as a non-adaptive and impulsive consequence of loss aversion and frustration (Eben, Chen, et al., 2020). However, we recently observed post-loss speeding in a task that did allow model-based learning and rational adaptation, as well as voluntary action choice: using probabilistic reversal learning (PRL), Majchrowicz et al. (2020) observed a numerical trend towards reduced reaction times (RTs) following losses compared to wins, suggesting a stronger urgency to act after losses. Interestingly, in the same task, losses led to a higher sense of agency over the trial following a loss. This agency boost has been associated with the individual tendency to learn from losses (Di Costa et al., 2018).

To further investigate post-loss speeding, the present study specifically focussed on RTs in a PRL task, and their electrophysiological correlates. We investigated whether losses, compared to wins, affected subsequent neurophysiological processes of motor preparation (readiness potential [RP]) and outcome evaluation (feedback-related negativity [FRN] and P300). Overall, we were interested in demonstrating post-loss speeding in a context that allowed the rational adaptation of action choices, which characterises learning. This could hint towards a potentially adaptive function of speeding in learning, rather than being merely the frustrated reaction to reward omission.

### Post-loss speeding in gambling tasks

Historically, *post-loss speeding* (PLS) was primarily observed in gambling tasks. In gambling, objectively, no adaptive response strategy can be found or exploited since gambles are independent chance events. In their seminal paper, Verbruggen et al. (2017) let participants choose between a certain monetary reward (non-gamble) or a gamble with known outcome probabilities. Once participants selected the gamble, they did not influence its outcome. When participants gambled and lost, their RTs to initiate the following trial became faster compared to trials in which they gambled and won or selected the non-gamble. The authors interpreted this speeding as an emotional effect arising from frustration and impulsivity. The invigoration of subsequent behaviour was assumed to help participants to quickly overcome an aversive emotional state, thus constituting a form of avoidance behaviour (even though the subsequent event was behaviourally approached). Losses also influenced RTs in a perceptual decision-making task that was intermixed with the gambling task, suggesting that speeding arose from low-level reactive and potentially motoric processes. However, an intermediate task between two gambles did not entirely cancel the propagation of speeding until the subsequent gamble, which speaks for some task sensitivity of the speed-up effect.

Following up on these findings, Eben et al. (2020) replicated PLS in comparable gambling tasks in which participants knew the outcome probabilities of a gamble but could not adaptively increase their gambling success. Losses not only increased subsequent speed but also the likelihood to gamble again. These findings are reminiscent of *loss-chasing* in gambling (Zhang & Clark, 2020), which is assumed to arise from compromised inhibitory control, mood-related impulsivity and compulsivity. In a series of experiments, Eben et al. (2020) further ruled out that speeding stemmed from attentional control arising from outcome novelty or expectancy (but see Chen et al, 2020). The results were taken to further support the *frustrative non-reward account*, suggesting that losses evoked a mismatch between desired and attained outcome-states, leading to discomfort. This aversive state reduced inhibitory control and led to an urgent, that is, uninhibited, exhibition of the most salient motor action plan (Frijda, 2010). Based on the framework proposed by the authors, increased urgency to act is expected to correspond with increased motoric salience of the subsequent action (Eben, Billieux, et al., 2020). Accordingly, the account predicts a more prominent motoric representation of actions following losses than those following wins.

Interestingly, Eben et al. (2022) observed larger PLS in an ‘illusion of control’ situation among participants who *believed*, incorrectly, that they influenced the outcome of a coin toss. The mere illusion of control was associated with speeding after losses, although this effect was small and short-lived. This hints towards a potential relationship between PLS and the subjective sense of agency. However, a follow-up experiment could not replicate this association, and the relationship between speeding and subjective sense of agency remains unclear. Some theoretical accounts predict the opposite direction of causation, namely that increased speeding can re-establish a *compromised* sense of agency in order to avoid perceived helplessness and resulting apathy and fatigue (Saunders et al., 2016). Indeed, helplessness in a frustrating task without control over the outcome led to stronger PLS (Dyson et al., 2018; Eben et al., 2023). At this point, the relationship between sense of agency and the invigoration of behaviour after losses still remains poorly understood.

### Probabilistic reversal learning

PRL provides an interesting paradigm to investigate the relation between PLS, learning, and subjective sense of agency. In PRL, participants repeatedly choose between two options which have different reward probabilities. Importantly, the reward mapping unpredictably switches, so that the previously optimal option becomes suboptimal. To keep rewards high, participants must continually monitor the outcomes of their actions and guide their behavioural choices accordingly. PRL thus allows and invites adaptive control and model-based optimisation of behaviour (Cools et al., 2002). While gambling is characterised by a succession of independent chance events, in which previous outcomes cannot be adaptively employed to rationally optimise future action, PRL clearly allows learning from experience. At the same time, both tasks involve uncertainty regarding outcomes. Therefore, PRL is a suitable candidate task for exploring PLS in a context that allows more agentic control than gambling but still resembles its phenomenological experience.

Previous PRL studies showed an increase in subjective sense of agency after losses compared to wins, an effect labelled *post-error agency boost* (PEAB; Di Costa et al., 2018). Losses indicate that the participants’ model of the world might require re-evaluation. Such re-evaluation and behavioural adaptation can, in turn, increase participants’ success, boosting objective performance, and, potentially, subjective sense of agency (Metcalfe & Greene, 2007). Importantly, losses did not increase agency in a random outcome condition where action-outcome learning was impossible, ruling out that PEAB arose from outcome uncertainty or the mere frustration upon encountering a loss.

In two experiments, Majchrowicz et al. (2020) related the PEAB effect to FRN and P300 event-related potential (ERP) components evoked by outcome presentation, which are correlates of belief-updating (Bennett et al., 2015). Interestingly, the authors observed a numerical trend towards speeding after losses compared to wins, but did not consider its relationship with PEAB or outcome monitoring. However, their method interrupted outcome-based action choices by introducing an interspersed time interval judgement task after every PRL trial, which might have dispersed or altered the processes involved in PLS. Nevertheless, these preliminary findings invite the speculation that PEAB and speeding co-occur, and that both are related to outcome monitoring, and thus potentially play a role in model-based learning.

### Aim of the present study

The present study focuses on three research questions. First, and most importantly, we investigated whether PLS occurred in a PRL task. This would extend PLS from the original context of gambling to contexts that involve model-based learning and adaptive behavioural control. Such findings would open the possibility that PLS might play a role in adaptive cognition, rather than merely representing affective avoidance as previously suggested by the *frustrative non-reward account*.

Second, we investigated whether previous losses affected the neurophysiological signature of motor preparation of the subsequent trial. Here we targeted the RP, a well-established physiological correlate of motor preparation of self-initiated movement (Kornhuber & Deecke, 1965; Schurger et al., 2021; Travers et al., 2020). If losses – as suggested by Eben et al. (2020) – indeed increased the motoric urgency to act in the following trial, we might expect higher RP amplitudes following losses compared to wins. However, if speeding after losses did not stem from a predominantly motoric effect, previous outcome valence should not influence the subsequent RP.

The third aim was to investigate whether previous losses affected neurophysiological outcome evaluation processes of the subsequent trial. In line with Majchrowicz et al. (2020), we focussed on the FRN and the P300. The FRN is a fronto-central ERP component peaking around 250 ms after feedback presentation (Nieuwenhuis et al., 2004; San Martín, 2012). In PRL tasks, the FRN is thought to encode reward prediction errors, that is, the discrepancy between expected and received outcomes (Chase et al., 2011; Holroyd & Coles, 2002). FRN amplitudes are typically more negative following negative feedback or unexpected outcomes, emphasising its role in updating reward expectations (Gehring & Willoughby, 2002). The FRN is less related to immediate behavioural adjustments, suggesting a broader role in signalling error detection rather than guiding actions (Glazer et al., 2018; Hauser et al., 2014; Von Borries et al., 2013). The FRN has also been related to post-decisional monitoring and agency evaluation (Bellebaum et al., 2010; Sidarus et al., 2017) and the boost in sense of agency that occurs after self-initiated actions that result in losses (Majchrowicz et al., 2020). To our knowledge, only this latter study investigated FRN effects associated with previous (rather than current) outcome values, demonstrating less negative FRN amplitudes in trials preceding losses compared to wins. Temporally following the FRN, the P300 component is a central positive deflection peaking between 300 and 500 ms after feedback presentation. It has been linked to various cognitive functions depending on task design (Polich, 2007). In decision-making and learning tasks, it reflects the categorisation of current outcome information, but has also been implicated in agency judgements (Kühn et al., 2011; Majchrowicz et al., 2020). In contrast to the FRN, the P3 amplitude is more influenced by the magnitude and salience of the feedback, reflecting the integration of outcome information for subsequent decision-making (Chase et al., 2011; Eppinger et al., 2008; Von Borries et al., 2013). Both the FRN and P3 are modulated by expectancy violations during reversal learning. Our hypothesis that PLS is linked to model updating predicts that these components should be modulated by previous losses.

## Method

### Participants

Twenty-five (18 female) participants aged 18 to 33 years (*M* = 20, *SD* = 4) participated in the task. Sample size was determined a priori based on the PEAB effect size obtained by Di Costa et al. (2018), *d* = 0.96 (large effect size; Cohen, 1988), power = 0.8 and, α = .05 for a 2 × 2 × 2 ANOVA using G*Power 3.1 (Faul et al., 2009). All participants were right-handed, had normal or corrected-to-normal vision, and had no history of psychiatric or neurological disorders. Participants gave written informed consent and were compensated at £7.50/hr plus and additional bonus up to £2.50 depending on their performance. The study was approved by the UCL Research Ethics Committee and carried out in accordance with the Declaration of Helsinki. This study was not preregistered.

### Stimuli and procedure

**Figure 1.**
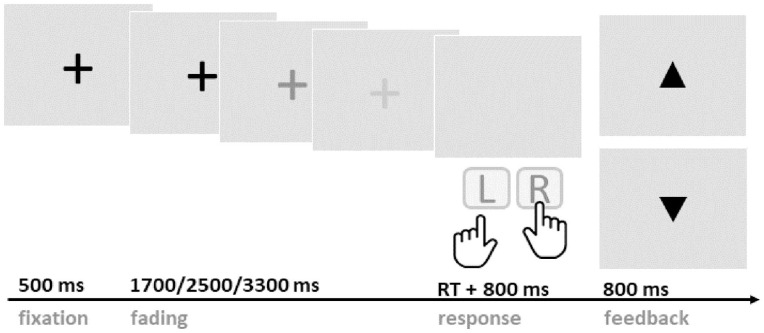
Schematic illustration of the probabilistic reversal learning paradigm. Participants were asked to wait, at least, until the fixation cross completely faded to respond with either the right or left key. Either an upward or downward pointing triangle was presented following the key press to indicate a win or a loss, respectively.

A PRL paradigm was adapted from Di Costa et al. (2018) and customised to visual presentation suited for EEG recording (see Figure 1). The experiment was programmed with Psychophysics Toolbox v3 (Brainard & Vision, 1997) and was run on Matlab (MathWorks, Natick, MA, USA) on a Windows desktop computer.

Participants were seated in a quiet room approximately 50 cm away from the computer screen. Throughout the experiment, participants were making choices between two buttons, either the left or right keyboard key. At every trial, one of the keys (the ‘correct’ key) delivered rewards with 80% and losses with 20% probability, while the other key (the ‘incorrect’ key) had reversed outcome probabilities. The participants’ task was to identify the correct key and to maximise their reward over time. Participants were informed that the correct key would stay the same for a while but then switch after a random number of trials. They were instructed not to try to anticipate such reversals. Unbeknownst to the participants, reversals occurred after the correct button was pressed consecutively five to seven times (randomised).

On every trial, a black fixation cross appeared in the centre of the light-grey screen, staying at full luminance for 500 ms and fading out at different rates (1,700, 2,500, or 3,300 ms). Jittered fading durations were introduced to avoid anticipation of a predictable response time. Participants were instructed to press either the left (F) or right (J) keyboard key with the index finger of the respective hand. Button presses were only accepted after the fixation cross had faded completely; premature responses led to a penalty of five points and trial abortion. After the key press, the screen remained blank for 800 ms, then the outcome was presented for 800 ms. Win and loss outcomes were represented as upward or downward pointing triangles, respectively. Participants received one point for each win, and no points for losses. If their performance within one block was above 60% (72 points), participants received a 50p bonus, allowing them to earn a maximal additional reward of £2.50. The main EEG task consisted of five blocks of 120 trials each, with breaks between blocks. Each block lasted around 10 min.

After the undisturbed PRL within the EEG blocks, participants completed one additional block in which intentional binding (IB) was measured. The aim of this block was to establish a link between the current experiment and previous findings by (Di Costa et al., 2018) and Majchrowicz et al. (2020). In particular, we were expecting to replicate the finding of a PEAB in the present adaptation of the PRL task. The IB block was similar to the main experiment, except for two adaptations: the duration between button press and outcome presentation varied between three durations (100, 500, 900 ms), and a tone accompanied the visual outcome presentation. Both wins and losses were indicated by the same tone presented by speakers. After each trial, participants were asked to estimate the time interval between their key press and the tone indicating outcome presentation. Reports were made verbally by the participant and written down by the experimenter. A practice period allowed participants to get accustomed to the intervals of 100, 500 and 900 ms, respectively. No monetary reward was given in the IB block. After this last block, participants were debriefed and received their reimbursement.

### EEG recording and pre-processing

In the main experimental session, electrophysiological activity was recorded using a BrainVision Recorder (Brain Products, Gilching, Germany) with 25 active Ag/AgCl electrodes set up on an ActiCap (Brain Products) according to the 10/20 system. Included electrodes were F3, Fz, F4, FC1, FCz, FC2, C3, C1, Cz, C2, C4, CP1, CPz, CP2, P3, Pz, P4, O1, Oz, O2, TP9, TP10, Fp1. Electrodes for vertical electrooculogram were placed above and below the right eye; horizontal electrooculogram electrodes were placed on the outer canthi of both eyes. Electrode impedance was held below 10 kΩ. The EEG signal was sampled at 500 Hz. Data was recorded with online reference to POz and re-referenced offline to the mean of left and right mastoids.

EEG recordings were analysed using EEGlab for Matlab (Delorme & Makeig, 2004) at the pre-processing stage and R at the statistical analysis stage. During pre-processing, raw EEG data were re-referenced to the mastoids average. The signal was filtered at 0.001 to 20 Hz, with the high cut-off during recording at 140 Hz, and downsampled to 256 Hz. Next, the data were subjected to an independent component analysis to remove eyeblinks and other typical artefacts. ICA-derived components were inspected and manually cleaned to remove eyeblinks and other typical non-brain artefacts. Data were then epoched relative to the events of interest. For outcome-locked analyses, the time selected window was −0.2 to 2.8 s. For action-locked analyses, the time window was set to −1 to 3 s. Then, automatic EEGlab algorithms were used for epoch cleaning. Finally, data were exported to R for visualisation (using the *ggplot2* package; Wickham et al., 2024) and statistical modelling using linear mixed effects models (using the *lme4* package; Bates et al., 2015).

### Statistical analysis

Modelling of both behavioural and EEG data was done with linear mixed effects models. The model selection procedure for each analysis consisted of a comparison of the model’s fit, starting with a maximal fixed term structure (i.e. including all potential factors of interest as fixed effects) and comparing it to more minimal versions of the fixed term (i.e. removing the factors based on their theoretical importance). After establishing the optimal fixed effects, the random effect’s structure was tested, starting with intercept-only, and then adding factors (in the order based on their theoretical importance) up to the stage when models no longer converged. In the results section, for each analysis, we report the following statistics for the winning models: estimate of the effect (β), *t*-value, *p*-value and 95% confidence interval (CI). In all models, contrast coding (−0.5/0.5) of dichotomous predictors was applied. Full formulas of the models are reported in the online Supplemental material.

### Transparency and openness

To comply with level two of the Transparency and Openness Guidelines by the Center for Open Science (Nosek et al., 2015), aggregated ERP data and processing code to reproduce the analyses have been made available through the OSF repository and can be accessed here: https://osf.io/c27wu/. Furthermore, all used program code and methods developed by others that were used are cited accordingly. The study design and analysis plan have not been pre-registered. According to the 21-word statement, we report how we determined our sample size, all data exclusions, all manipulations, and all measures that were collected in the study.

## Results

### Behavioural results of main EEG blocks

#### Accuracy

For the analysis of the five EEG blocks, one participant who did not perform significantly above chance level (51% accurate button presses) was excluded. For the remaining 24 participants, performance was significantly above chance, with 72% correct button presses resulting in 64% winning outcomes, indicating successful learning. On average, 47 reversals occurred throughout the five blocks of the main experimental session, with an average of 12 trials per mapping. Anticipatory responses, that is, invalid key presses initiated before the fixation cross had completely faded, occurred in 0.8% of the trials and were removed from the analyses. Of all premature responses, 50 occurred after wins, 64 after losses, and 5 following undefined outcomes (e.g. block transitions or aborted trials). To achieve normal distribution, RT data was log transformed, and outliers (±3 *SD*) were removed, which led to the removal of 1.2% of all trials.

#### Reaction times

##### Effect of previous outcome valence

The main aim of the present study was to investigate PLS in the context of PRL. We observed significant PLS in the EEG blocks. The model revealed a significant effect of previous outcome valence (β = .07, 95% CI = [0.03–0.11], *t* = 3.41, *p* < .01), indicating that participants were faster to respond in trials following losses (708 ms) than wins (758 ms). We scrutinised this result by further employing the robust Dutilh method (Dutilh et al., 2012), which only compares the difference in RTs between post-error trials and their associated pre-error trials. Using 23.6% of all trials, the robust method confirmed that RTs following losses were significantly faster (615 ms) than following wins (671 ms; *t* = 3.93, *p* < .001).

##### Response strategy

We then investigated whether PLS was affected by the subsequently chosen response strategy, that is, whether participants stayed with the same button or switched buttons. For a meaningful analysis we had to selectively focus on behaviour following losses, since participants acted rationally and almost never switched their responses following wins. Selecting the subset of data in which the previous outcome was a loss, our model did not show a significant effect of response strategy (β = .02, 95% CI = [−0.03 to 0.07], *t* = 0.9, *p* = .37). As such, losses led to faster responses, regardless of whether participants chose to press the same button (660 ms) or switched button (715 ms).

### Behavioural results of IB block

#### Accuracy

For the analysis of the IB block, two participants who did not perform significantly above chance level (51% and 56% accurate button presses, respectively) were excluded. For the remaining 23 participants, performance was significantly above chance, with 70% correct button presses resulting in 64% winning outcomes. On average, nine reversals occurred throughout the IB block, with an average of 13 trials within one mapping. Anticipatory responses, i.e., invalid keypresses that occurred before the fixation cross had faded completely, were observed in 2% of the trials. To achieve normal distribution, RT data was log transformed, and outliers (±3 *SD*) were removed, which led to the removal of 1.5% of all trials. Further removing deviant interval estimates (±3 *SD*) led to the additional deletion of 0.7% of trials.

#### Interval estimations

##### Effect of previous and current outcome valence

**Figure 2.**
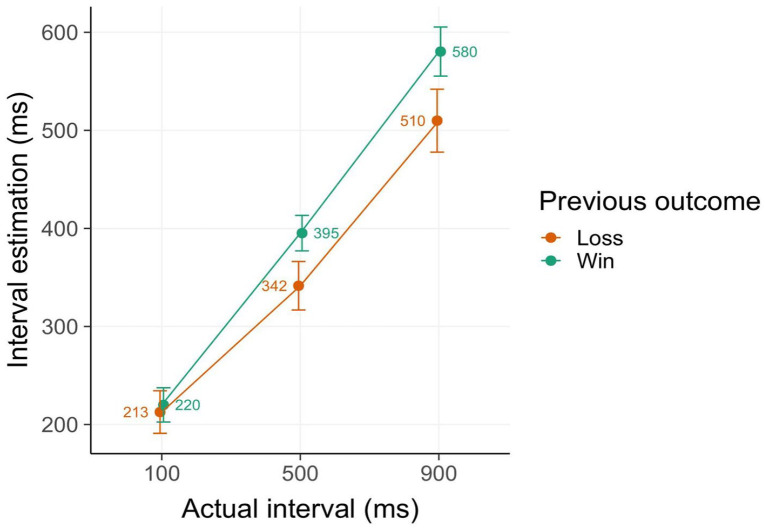
Intentional binding results. Interval estimates (in ms) are shorter in trials following losses compared to wins, indicating an increased sense of agency following losses, the so-called *post-error agency boost* effect. Error bars denote 2 SE.

To replicate the PEAB effect, that is, shorter interval estimates (corresponding to increased sense of agency) following losses, a model including effects of previous and current losses was fitted (cf. Majchrowicz et al., 2020). We observed a strong main effect of actual interval (β = 135.72, 95% CI = [100.40–171.04], *t* = 7.53, *p* < .001) as well as of previous outcome (β = 34.2, [17.87–50.53], *t* = 4.11, *p* < .001) (Figure 2). No other effects or interactions were significant. These results demonstrate that interval estimations were affected by previous outcome valence, with shorter temporal estimations (thus higher sense of agency) following losses compared to wins. This finding replicates the PEAB effect observed in similar versions of the reversal learning task (Di Costa et al., 2018; Majchrowicz et al., 2020).

##### Response strategy

We investigated whether IB after losses was affected by the subsequently chosen strategy to respond to the loss (stay with the same button vs. switch to the other button). For a meaningful analysis, the subset of data in which the previous outcome was a loss was selected, as switches after wins almost never occurred. The model did not show a significant effect of response strategy (β = 19, 95% CI = [−21.23 to 59.22], *t* = 0.93, *p* = .35). Thus, similar to RT, IB after losses was not affected by the selected behavioural response strategy. Since the response strategy did neither affect RT in the EEG block nor IB in the IB block, we excluded this variable from further analyses.

#### Relationship between IB and RT

Since both RT and IB were affected by previous outcome valence, we investigated whether both measures also showed a direct relationship.

##### Influence of IB on subsequent RT

First, we tested whether the amount of implicit agency experienced during the previous trial (as indexed by IB) affected how quickly the following trial was initiated. In line with previous analyses, the fitted model revealed a significant main effect of previous outcome on current RT (β = .08, 95% CI = [0.03–0.14], *t* = 2.89, *p* < .005). The effect of previous IB on current RT was marginal, reaching significance in the model without random slopes of IB (β = .03, [0.01–0.05], *t* = 2.5, *p* < .05), but falling out of significance when random slopes of IB were included (β = .03, [−0.00 to 0.06], *t* = 1.81, *p* = .071). Model comparisons suggested that the former model provided a better fit only marginally (and the chi square test for the difference was not significant). Thus, while stronger IB (that is, shorter perceived intervals) might be associated with faster RT in the subsequent trial, this effect is not robust. The interaction term between previous outcome and previous IB was not significant (β = −.01, [−0.05 to 0.04], *t* = −0.32, *p* = .75).

##### Influence of RT on IB

Next, we tested whether the time required to make a button press (i.e., RT) affected the amount of agency experienced over that button press (IB). The fitted model showed a main effect of previous outcome (β = 36.69, 95% CI = [16.90–56.48], *t* = 3.64, *p* < .001), in line with the previous analysis. The relevant main effect of RT and the interaction effect were not significant (RT: β = −8.35, [−33.81 to 17.11], *t* = −0.64, *p* = .52; interaction: β = −.29, [−26.87 to 26.30], *t* = −0.02, *p* = .98). Thus, acting faster did not lead to an increased sense of agency within the same trial.

### EEG results

#### Action preparation: RP

**Figure 3.**
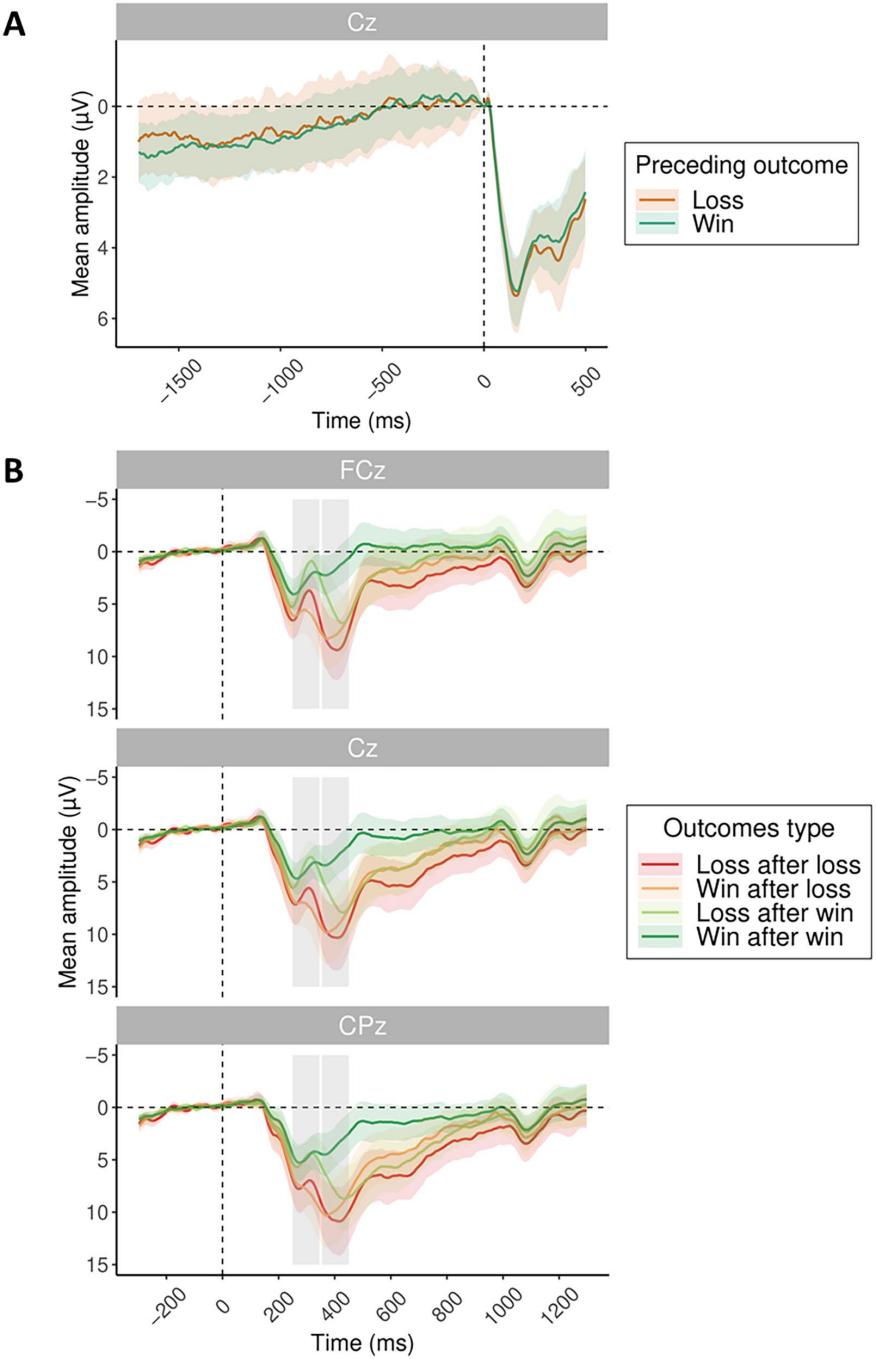
ERP results. (A) Readiness potential at Cz time-locked to action onset (button press) split by previous outcome valence: previous loss (orange) and previous win (turquoise). (B) Grand average ERPs time-locked to outcome presentation, split by previous outcome and current outcome valence, with FRN and P3 time windows marked by grey rectangles. *Note*. FRN = feedback-related negativity.

The second aim of the present study was to investigate whether the RP during preparation for subsequent action would differ depending on previous outcome valence. As losses lead to shortened RT and heightened sense of agency in the subsequent trial, we targeted the RP as a potential marker of differences in the build-up of an urgency to act following losses. For this, the action preparation phase was extended by presenting a fading fixation cross for at least 1,700 ms before subsequent action was possible. Baseline was set to −5 to 5 ms relative to the action onset (as in Khalighinejad et al., 2018). We analysed the time window of action preparation separately for trials following losses and wins. The effect of previous outcome valence on average amplitudes at electrode Cz in the 1,700 ms time window prior to action onset (button press) was entered in a model. However, the factor of previous outcome valence did not show any significant effect (β = .14, 95% CI = [−0.39 to 0.67], *t* = 0.54, *p* = .59) during subsequent action preparation. Thus, motor preparation as indexed by RP following losses did not show a distinct pattern from wins in the way RT and IB did (Figure 3A).

#### Outcome processing: FRN and P300

The third aim of the present study was to investigate the ERPs related to outcome processing and explore their potential association with PLS. Figure 3B shows the grand average ERP split by previous and current outcome valence at electrodes FCz, Cz and CPz.

#### Feedback-related negativity

##### Effect of previous and current outcome valence

**Figure 4.**
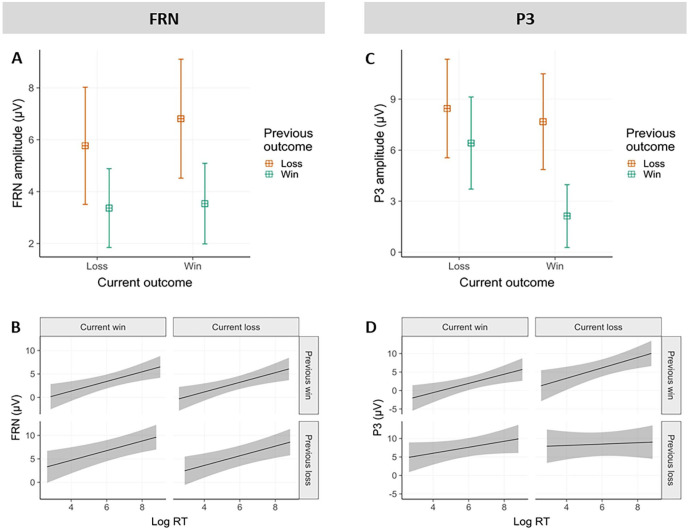
EEG results. (A) FRN as a function of previous and current outcome valence; (B) FRN as a function of current RT, previous and current outcome valence; (C) P300 as a function of previous and current outcome valence; (D) P300 as a function of current RT, previous and current outcome valence; (A) and (C) show models’ predictions with 95% CI, (B) and (D) shows actual data averages with 2 SEs. *Note*. FRN = feedback-related negativity; RT = reaction time.

Based on visual inspection, the average amplitude of the FRN was determined in a 250 to 350 ms outcome-locked time window, and pooled over electrodes Fz, FCz, Cz and CPz. Modelling revealed a significant main effect of previous outcome valence (β = −2.84, 95% CI = [−4.00 to −1.68], *t* = −4.78, *p* < .001) and an interaction between previous and current outcome valence (β = −.87, [−1.64 to −0.1], *t* = −2.22, *p* < .05). The effect of current outcome valence was not significant (β = .61, [−0.17 to 1.38], *t* = 1.53, *p* = .13). FRN amplitudes were less negative in trials following previous losses compared to wins. This result replicates the finding that in free choices, previous losses were associated with less negative FRN amplitudes at current outcome presentation compared to wins (Majchrowicz et al., 2020). The interaction indicated that if the previous outcome was a loss, FRN did hardly discriminate between current wins and losses, whereas when the previous outcome was a win, current losses were associated with less negative FRN amplitudes compared to current wins (see Figure 4A).

##### Relationship between RT and FRN within the same trial

Importantly, to test the influence of PLS on FRN amplitudes, we investigated whether RTs had a direct effect on FRN amplitude elicited by feedback presentation within the same trial. As already observed, previous losses were associated with less negative FRN amplitudes compared to previous wins (main effect of previous outcome: β = −2.89, 95% CI = [−4.05 to −1.74], *t* = −4.93, *p* < .001). Interestingly, independent of this main effect of previous outcome, a main effect of RT emerged (β = 1.03, [0.38–1.68], *t* = 3.1, *p* < .01), indicating that faster RTs were associated with more negative FRN amplitudes within the same trial, across all outcome conditions (see Figure 4B). We again observed a significant current-by-previous outcome interaction (β = −.91, [−1.68 to −0.14], *t* = −2.32, *p* < .05) as described in the previous section.

#### P300

##### Effect of previous and current outcome valence

The P3 component was computed across a 350 to 450 ms outcome-locked time window, and pooled over electrodes Fz, FCz, Cz, CPz and Pz. Modelling showed a significant main effect of previous outcome (β = −3.79, 95% CI = [−5.01 to −2.57], *t* = −6.09, *p* < .001), current outcome (β = −2.53, [−3.70 to −1.37], *t* = −4.26, *p* < .001), and an interaction between previous and current outcome (β = −3.52, [−5.07 to −1.97], *t* = −4.45, *p* < .01). The P3 amplitude was more positive following both previous and current losses compared to wins. The interaction indicated that if the previous outcome was a win, P3 amplitude distinguished between current outcomes. However, if the previous outcome was a loss, current outcome had no influence on P3 amplitudes. This interaction pattern is somewhat similar to the one observed for the FRN (see Figure 4C).

##### Relationship between RT and P300 within the same trial

We further investigated the direct relationship between RT and P3 amplitude within the same trial, in addition to the factors of previous and current outcome valence. Both main effects of RT and previous outcome and their interaction were significant. As already observed, P3 amplitude was more positive following previous losses compared to wins (β = −9.01, 95% CI = [−13.68 to −4.33], *t* = −3.77, *p* < .001). Longer RTs were associated with more positive P3 amplitudes elicited by the presentation of that action’s outcomes (β = .91, [0.12–1.7], *t* = 2.26, *p* < .05). Furthermore, a significant interaction (β = .83, [0.11–1.55], *t* = 2.26, *p* < .05) indicated that longer RTs resulted in more positive P3 amplitudes especially after previous wins, but not as much after previous losses. No other effects were significant (see Figure 4D).

## Discussion

The aim of the present study was to investigate three questions: (a) Do losses in PRL lead to faster subsequent responses compared to wins, that is, can we observe PLS in the context of learning? Furthermore, does previous outcome valence affect ERPs of the subsequent trial, in particular (b) the neurophysiological correlate of motor preparation (RP) of the subsequent button press and (c) the neurophysiological signature of subsequent outcome presentation (FRN and P300)? Especially the last question aimed to explore the possibility of an adaptive role of PLS in the context of PRL.

### Do losses in a PRL task lead to faster subsequent responses compared to wins?

First, our results clearly demonstrate faster response times following losses compared to wins in the employed PRL task. We thus show evidence of PLS in the context of learning, rather than gambling. This speeding effect co-occurred with the previously described PEAB effect, that is increased sense of agency after losses compared to wins (Di Costa et al., 2018; Majchrowicz et al., 2020). Neither PLS nor the PEAB effect was influenced by the decision of participants whether to stay with the same button or to switch buttons after a loss. This might be taken as a first hint that speeding was not related to motor aspects of action selection or execution. This finding replicates the previously observed dissociation between behavioural choice and action speed (Verbruggen et al., 2017) and resonates with findings of absent influence of action selection fluency on agency (Sidarus et al., 2017). Our design further allowed us to investigate the direct relationship between PLS and PEAB in a subset of the data. Here, a stronger sense of agency measured by IB was associated with faster RTs in the subsequent trial. However, this effect was not robust, and we refrain from its interpretation. Similarly, faster action initiation did not influence the agency perceived over that action. These results do not support a direct relationship between PEAB and post-loss speeding.

### Do losses in a PRL task affect the RP preceding the subsequent button press?

The second aim of the study was to investigate whether previous outcome valence affected the neurophysiological correlate of motor preparation of the subsequent action as predicted by the *frustrative non-reward account* (Eben, Billieux, et al., 2020; Frijda, 2010). As previous losses led to faster RTs and a heightened sense of agency in the subsequent trial compared to wins, we targeted the RP as a differential marker of the build-up of urgency to act following losses. However, we did not observe an effect of outcome valence on RP amplitude. As such, the increased urgency to act after losses seems unrelated to the motoric preparation of the subsequent action as indicated by RP. This finding is in line with the above observation that PLS was not affected by the selected motor response.

### Do previous losses in a PRL task affect the neurophysiological signature of outcome evaluation?

The third aim of the present study was to investigate the influence of previous outcome valence on ERPs related to the presentation of the following trial’s outcome. With regard to the FRN, we observed fewer negative amplitudes following previous losses compared to wins. If the previous outcome was a win, the current outcome valence showed a similar effect as the previous outcome valence, namely, less negative FRN amplitudes elicited by current losses compared to wins. While the observed effect of previous outcome valence on FRN replicates a previous finding (Majchrowicz et al., 2020), the FRN effect of current outcome is at odds with reports that losses, especially if less frequent than wins, lead to more negative FRN amplitudes compared to wins (Dyson et al., 2020). Different calculation methods could be discussed as potential reasons for discrepant findings. For instance, different time intervals or methods of signal isolation might have been more sensitive to negative deflections following losses at around 300 ms. Furthermore, it seems important for future studies to investigate earlier ERP components affected by previous outcome valence. Based on visual inspection, the factor of previous outcome shows an early valence differentiation with a positive peak around 250 ms, preceding the FRN time window. Future studies could target what we here retrospectively interpret as a reward positivity, a positive deflection in response to rewards and reward cues with a central topography, which has been implied in learning processes and is elicited by striatal reward areas (Cockburn & Holroyd, 2018; Glazer et al., 2018). It could be assumed that more positive FRN values following previous losses versus wins are potential carry-over effects of an earlier occurring effect of reward positivity. Taking such an effect at face value would suggest that outcomes following previous losses are processed as more rewarding compared to outcomes following wins. We will come back to this interpretation when discussing the potential function of PLS. Investigating the direct relationship between action initiation speed and FRN amplitude at outcome presentation showed that faster RT were associated with more negative FRN amplitudes within the same trial, a pattern that was consistent across all valence conditions.

With respect to P300 amplitudes, previous losses led to generally more positive amplitudes compared to wins, thus showing a similar pattern as observed for the FRN. Similar to the FRN, current losses resulted in more positive P3 amplitudes compared to wins if the previous outcome was a win, but not when the previous outcome was a loss. Taking into account the influence of RTs on P300 amplitudes showed that slower responses were associated with more positive amplitudes elicited by that action’s outcome. However, this pattern was most pronounced after previous wins, and almost absent following losses.

Overall, the patterns of FRN and P3 showed some overlap but also differences. For instance, while RT had an effect on FRN across all conditions, this linear effect on P3 was limited to previous wins. It has to be noted that the P3 occurs in high temporal and spatial proximity to the FRN, so it cannot be ruled out that FRN effects were carried over and affected P3 amplitudes. Even in this case, however, the present data suggest a functional dissociation of FRN and P3 with regard to current outcome valence monitoring (Wurm et al., 2022), to which P3 amplitudes were more sensitive. More positive P3 amplitudes have been associated with model updating and behavioural adjustment. However, just like the FRN, P3 amplitudes have been argued to be potentially influenced by preceding reward positivities (Glazer et al., 2018).

### PLS: does it have an adaptive function?

Taken together, our findings clearly demonstrate a PLS effect in PRL. This effect seems unrelated to motor preparation and execution but possibly interacts with neurophysiological processes of outcome monitoring and evaluation. An interesting novel finding of our study is the direct relationship between RT and FRN amplitude, with faster RTs associated with more negative FRN amplitudes elicited at outcome presentation.

Compared to gambling tasks, PRL allows learning, characterised by exploring and exploiting models of the environment by repeated interaction. However, the task also shares characteristics with gambling, including the need to initiate actions (behavioural choice) which lead to rewards or losses (valenced outcomes and action-outcome contingency, that is, a form of agency) which are not entirely predictable (uncertainty). In fact, a well-known bias in gambling arises in the context of these shared characteristics. The *illusion of control* describes the bias to overestimate the likelihood of favourable outcomes of random events if an action can be chosen freely rather than being externally imposed (Langer, 1975). These overlapping characteristics raise the important question of whether PLS occurs in PRL because PRL resembles gambling, or PLS occurs in gambling because gamblers tend to wrongfully assume that some learning and adaptation is possible in a game involving the succession of independent chance events.

Compared to independent chance events, the repeated possibility to test an action-outcome sequence might constitute the most important difference between contexts of learning and gambling. However, this feature can also be erroneously ascribed to gambling contexts, as demonstrated by the *gambler’s fallacy*. This bias refers to the erroneous assumption that outcomes of independent chance events (i.e. separate gambles) influence each other. For instance, assuming an anti-correlation, a streak of losses tends to be wrongfully assumed to increase the likelihood of a following win (Tversky & Kahneman, 1971). Biases like *illusion of control* and *gambler’s fallacy* demonstrate that behavioural tendencies which are adaptive in learning environments tend to spill over to gambling where they no longer serve their purpose. Thus, the speed-up observed in gambling might be based on a similar biased assumption that gambling events are not independent and, consequently, some adaptive signal can be found in the pattern of one’s actions’ outcomes.

Speeding could thus constitute a form of signal chasing. Previous findings show stronger PLS in gambles with low winning probability but potentially high rewards, corresponding with high uncertainty, a situation in which evidence is desirable. As an instance of evidence chasing, speeding up RTs might be interpreted as the increased effort to receive more information as quickly as possible. This resembles the effect that gamblers are willing to pay money to receive informative cues about the outcome of a gamble (Brydevall et al., 2018).

Furthermore, evidence can also be internal, represented by neurophysiological signal strength. The observed direct relationship between RT and FRN amplitude seems to suggest the interesting possibility that a neurophysiological signal can be behaviourally modulated. If speeding increases the FRN amplitude of the following outcome, it might be agentically employed to modulate some relevant internal physiological signal, be it for the sake of gathering relevant information or counteracting aversion (or both). This is an intriguing moment where action seems to affect perception. Such a link could allow participants to induce a potentially hedonic effect irrespective of objective outcome valence, simply by increasing reaction speed. The FRN is a correlate of prediction error that relies on repeated interaction with an environment that is not absolutely unpredictable with the goal of guiding future behaviour based on previous outcomes. Thus, speeding interacts with a neural marker of learning and model building (Bellebaum et al., 2010). Such modulation, in particular following losses, was not observed for P300 amplitudes. Taken together, such compensatory influence over one’s own neurophysiological response might re-establish agency while objective control over favourable outcomes is (temporarily) low. While this form of regulation might prove useful in the context of learning to sustain motivation and action, it also bears the risk of being detrimental in situations where nothing can be learned and perseverance increases negative consequences, just as it might be the case in gambling.

Our study cannot disentangle whether speeding increased hedonic or instrumental internal signals, as outcome feedback served both as a learning signal and as a monetary reward (Bennett et al., 2019). Furthermore, we know that information is in itself hedonic (Brydevall et al., 2018) and both, rewards and reward cues, activate similar dopaminergic pathways (Weismüller et al., 2019). The observation that in our data outcomes following losses showed a visual trend towards a reward positivity might suggest that these outcomes were perceived as more rewarding. In a similar vein, the fact that speeding was associated with more negative FRN amplitudes suggests that speeding most likely serves an appetitive function, rather than avoiding and attenuating the internal outcome information, which would have produced a more positive FRN signal (Beyer et al., 2017). In this sense, speeding seems to increase the internal trace of an internal hedonic signal and could thus affect how we implicitly process our action’s outcomes.

### Limitations and outlook

It has to be noted that the ideas discussed above are post-hoc speculations. The observed relationships are purely correlational, and no causal directionality between speeding and FRN can be inferred. Our results cannot rule out the possibility that RT has no direct causal influence on FRN or that the observed relationship is driven by another latent factor that cannot be agentically modulated. One candidate for such a common factor is subjective time compression (Hoerl et al., 2020). Both shorter RTs and previous losses increased the sense of agency and might be two sides of subjective time compression after a loss. If, after a loss, all occurring events are speeded up in subjective time, subjectively contracted time would lead both to shorter time estimates (higher IB) and invite faster (re)action, thus speeding up RT. More generally, an interplay between different cognitive mechanisms might account for speeding. More research is needed to better understand the phenomenon of PLS and its relationship with agency, outcome processing and learning. For instance, future studies might employ computational models to relate RT with individual learning success and employed learning signals as done by Di Costa et al. (2018). Importantly, as previously mentioned, our data strongly invites further investigation of the relationship between PLS and earlier ERP components such as the reward positivity in PRL.

A recently presented account has suggested that what numerically looks like PLS might actually stem from the increase of RTs after wins, constituting what should be more adequately called *post-win slowing* (Dyson, 2024). In two experiments, the author demonstrated that in gambles (game of dice and paper-scissor-stone), RTs after wins varied as a function of the relative frequency of wins/losses and the presence of explicit win/loss labels, while RTs after losses did not show this variation. While overall RTs were slower after wins (i.e. faster after losses), this valence effect decreased with the relative increase of wins and was absent when explicit labels of outcome valence were not presented. Furthermore, the employed paper-scissor-stone game offered the outcome of a draw, constituting an outcome of neutral valence, which led to similar RTs as losses. This account offers an important alternative conceptualisation of post-loss slowing in gambling, inviting further research to disentangle the influence of wins and losses on subsequent RTs. Since our study did not include a valence-neutral baseline condition, we cannot rule out that the relative increase of response latencies after wins drives our PLS findings. Further studies should incorporate neutral baseline trials in addition to wins and losses.

Interestingly, while in the present study wins were more frequent than losses, RT after losses did not become slower compared to wins, as might have been predicted by the above account (Dyson, 2024; Dyson et al., 2018). Furthermore, while losses in the present study were not associated with a loss of points but with the absence of added points, our loss condition shares some similarity with a neutral draw condition. However, as only two outcomes were possible, the absence of a win most likely was interpreted as a negative loss outcome in our study. Nevertheless, our data might be relevant for the post-win speeding account, as it does not fully support some of its predictions.

## Conclusion

The present study set out to investigate PLS in the context of a task that allows behavioural adaptation and learning, namely PRL. Our results confirm faster RTs after losses than after wins, thus extending PLS from gambling to cognitive contexts where learning was possible and did occur. We additionally used EEG to investigate how losses influenced subsequent markers of action generation (RP) and outcome evaluation (FRN and P300). We found that previous losses did not affect the RP, suggesting that PLS was not primarily related to action preparation. However, compared to wins, previous losses lead to more positive FRN and P300 amplitudes elicited by subsequent outcomes. Interestingly, within-trial analysis revealed that faster RTs were associated with more negative FRN amplitudes within the same trial, irrespective of outcome valence. We discussed the possibility that PLS in PRL may represent a form of signal chasing, allowing participants to behaviourally modulate neurophysiological responses and thereby potentially establish agency by influencing internal neurophysiological signals.

## Supplemental Material

sj-docx-1-qjp-10.1177_17470218251333429 – Supplemental material for Post-loss speeding and neurophysiological markers of action preparation and outcome processing in probabilistic reversal learning Supplemental material, sj-docx-1-qjp-10.1177_17470218251333429 for Post-loss speeding and neurophysiological markers of action preparation and outcome processing in probabilistic reversal learning by Eugenia Kulakova, Bartosz Majchrowicz, Şiir Su Saydam and Patrick Haggard in Quarterly Journal of Experimental Psychology
