# Supplementary material for: Post-loss speeding and neurophysiological markers of action preparation and outcome processing in probabilistic reversal learning
Source: Q J Exp Psychol (Hove). 2025 Apr 2;79(1):87–101. doi: 10.1177/17470218251333429 (PMC12728083; doi:10.1177/17470218251333429)
Supplement: sj-docx-1-qjp-10.1177_17470218251333429 – Supplemental material for Post-loss speeding and neurophysiological markers of action preparation and outcome processing in probabilistic reversal learning [file sj-docx-1-qjp-10.1177_17470218251333429.docx]

**Supplementary materials**

**Post-loss speeding and neurophysiological markers of action preparation and outcome processing in probabilistic reversal learning**

Eugenia Kulakova, Bartosz Majchrowicz, Şiir Su Saydam, Patrick Haggard

***Model formulas.*** Model selection procedure based on comparison on models’ fit.

**3.1. Behavioural results of main EEG blocks**

***Reaction times***

Effect of previous outcome valence:
*Log RT ~ Prev. outcome + (Prev. outcome | Participant)*

Response strategy:
*Log RT ~ Button choice + (Button choice | Participant)*

**3.2. Behavioural results of intentional binding blocks**

***Interval estimations***

Effect of previous and current outcome valence:
*IB ~ Interval [rescaled] + Prev. outcome * Curr. outcome
 + (Interval [rescaled] + Prev. outcome * Curr. outcome | Participant)*

Response strategy:
*IB ~ Button choice + (Button choice | Participant)*

***Relationship between intentional binding (IB) and reaction time (RT)***

Influence of IB on subsequent RT:
*Log RT ~ Prev. IB [z-scored] * Prev. outcome + (Prev. outcome | Participant)* & *Log RT ~ Prev. IB [z-scored] * Prev. outcome + (Prev. IB + Prev. outcome | Participant)*

Influence of RT on IB:
*IB ~ Log RT * Prev. outcome + (Log RT | Participant)*

**3.3. EEG results**

***3.3.1. Action preparation: Readiness potential***

*RP amplitude ~ Prev. outcome + (Prev. outcome | Participant)*

***3.3.2. Outcome processing: FRN and P300***

***3.3.2.1. Feedback-related negativity (FRN)***

Effect of previous and current outcome valence: *FRN amplitude ~ Prev. outcome * Curr. outcome + (Prev. outcome + Curr. outcome | Participant)*

Relationship between reaction time and FRN within the same trial:
*FRN amplitude ~ Log RT + Prev. outcome * Curr. outcome +
(Log RT + Prev. outcome + Curr. outcome | Participant)*

***3.3.2.2. P300***

Effect of previous and current outcome valence: *P3 amplitude ~ Prev. outcome * Curr. outcome + (Prev. outcome * Curr. outcome | Participant)*

Relationship between reaction time and P300 within the same trial:
*P3 amplitude ~ Log RT * Prev. outcome * Curr. outcome +
(Log RT + Prev. outcome + Curr. outcome | Participant)*
